# 

**Published:** 1888-11

**Authors:** 


CONTENTS

Vol. 35 No. 11.

| | PAGE. |
|---|---|
| Special | 241 |
| Turning the Tables | 242 |
| Good Health by Right Living | 243 |
| Magnetic Hygiene | 247 |
| Divine Providence | 248 |
| The Oil Glands | 249 |
| A Case of Clairvoyance | 250 |
| Yellow Fever Germs | 253 |
| Sulphur for Medicinal uses | 254 |
| Administration of Acids, Alkalies, etc | 254 |
| Soap Making | 255 |
| What is Disease | 255 |
| Artificial Butter | 258 |
| Cramps in the Leg | 259 |
| Fate of an Epicure | 259 |
| Fatal Tight Lacing | 260 |
| Book Notices | 261 |
| Household | 262 |
| Miscellaneous | 263 |

INDEX TO ADVERTISEMENTS OF HALF A PAGE AND OVER.

| | PAGE. |
|---|---|
| Page & Co | I |
| Yankee Blade | I |
| Stevensville Mills | II |
| The Herb Cure Co | II |
| The Farm aud Household Cyclopædia | III |
| Hollow Suppositories | IV |
| Lactopeptine | V |
| The Family Cyclopædia of Useful Knowledge | VI |
| Lecture Programme of Prof. George Chainey | VIII |
| Rio Chemical Co | X |
| Davis' Elixir of Health | XI |

